# The BBC: Guardian of Public Understanding

**DOI:** 10.1007/978-3-030-51701-4_4

**Published:** 2020-11-13

**Authors:** Jean Seaton

**Affiliations:** 1 Department of Political Science, Leiden University, Leiden, The Netherlands; 2 School of Governance, Utrecht University, Utrecht, The Netherlands; 3 School of Governance, Utrecht University, Utrecht, The Netherlands; University of Westminster, London, UK

**Keywords:** BBC Broadcasting, British politics, News media, Institutional independence, Institutional crisis

## Abstract

The British Broadcasting Corporation (BBC) is a long-standing institution with a worldwide reputation as the maker and supplier of trustworthy news embedded in programmes aimed at serving the public not commoditising it. This chapter describes the BBC’s institutional DNA and explains how its birth characteristics informed its institutional trajectory over the decades. The chapter discusses the internal principles and the particular ‘craft’ that has made this a true British institution that has become equally revered outside Britain’s borders. It analyses how the BBC survived institutional crises to reach a moment in history where the very idea underlying this venerable but agile institution has come under fire.

## An Institution at the Crossroads

The British Broadcasting Corporation (BBC) has endured as institution over the decades, surviving many challenges. Launched in 1927 as a British public service institution that would ‘inform, educate and entertain’, it has become a British institution that served the world. It enriched democracy by serving audiences, irrespective of class, wealth, age and any other division, all over the nation, as equal citizens. It has served the world by reporting expertly and fairly into many closed and authoritarian political systems and by displaying cultural ingenuity and sensitivity in doing so. It has held power to account and represented the interests and voices of the less powerful. The founders saw the BBC as an institution that would create informed public opinion as the basis for functioning democracy at a time of risk. They thought the Corporation needed to accumulate authority and trust to be an independent arbiter of information. The first and founding principle was editorial independence. Yet the trust and reliability that this engendered had to be remade time and again throughout its history.

The BBC was designed to represent the nation to the nation, the world to the nation and the nation to the world. It is the epitome of the project of sceptical empirical inquiry that the Enlightenment proposed; with the imperative of finding, holding, amusing and serving audiences. But the BBC has also been challenged and threatened.

Finding new ways of securing the legitimacy of properly based information is a global challenge. The revolution in public understanding, the collapse in the legitimacy of evidence, the emergence of mis- and disinformation are new challenges. The BBC potentially remains an important tool, one of the few we have that is dedicated to the public interest, for re-engineering rational debate. The issue now is how to relate that reliability to everyone.

This chapter describes the institutional origins of the BBC and details its institutional DNA. It explains how this has informed the BBC’s institutional trajectory, helping it survive many political and societal challenges. It discusses the BBC’s current position in a fast-changing world where public institutions and the idea of fact-based news have come under fire.

## The Birth of the BBC

It is a fretful and uncertain time. There is concern that voters have insufficient knowledge to make rational judgements, in particular, that a new tranche of the electorate who have not previously voted will inadvertently vote irresponsibly because they will make choices on partisan or partial information. It is feared they may be easily misled. There is legitimate anxiety that hostile foreign powers are seeking to influence elections and political movements in novel ways.

There is a new technology barely understood by politicians and civil servants that they suspect may have influences they don’t trust: but frankly, most are completely out of touch with how it works or what it might be. So, they are quite unable to think through the opportunities and threats it poses. There is a distrust of the press which is seen as sensationalist and mendacious because of recent experience. There is a lack of trust in politicians (because they have apparently led the nation towards a variety of disasters). There is alarm about the detrimental impact ‘propaganda’ can have on behaviour, un-anchoring voting from authentic interests; a nagging worry that public opinion can be ‘bought’ by political interests; and an anxiety about the role of big business in politics. A political anxiety about the ‘people’ is mirrored by the possibility of revolutionary disruption.

The year is 1922. Creating the BBC is the solution to the problems sketched above. Of course, few understood that it was a solution at the time. The BBC was founded before anyone had the idea that broadcasting technology might matter to more than a few, techy ‘experimenters’. Besides, everyone knew that fun was nothing to do with the solemn business of government. Yet within a mere four years (from 1922–1926) Asa Briggs, the first great historian of the BBC wrote that it was remarkable that:

Broadcasting itself ceased to be a toy, an amusing novelty, an affair of ‘stunts’ and gimmicks: it became an institution. It affected people’s ways of thinking and feeling, and their relations with each other. (Briggs 1961: 4)

To understand how the BBC became so important one has to combine some sense of the inner life of the organization with that of the changing place of the organization in society. And this has to be situated within an international context. The BBC swiftly developed an international service and role that was both a reflection of UK values and a gift to the world, and other broadcasting systems came to be measured (and in some ways still are a century later) by reference to the BBC. And perhaps its significance had a philosophical basis as well. The BBC worked, because it was dedicated to a principal of reality: ‘an order of things that is independent of us, where that means, in particular, independent of our will’ (Williams 2002: 115).

Spreading broadcasting all over the nation as fast as possible, for the same ‘licence fee’ (in effect a hypothecated tax) paid by everyone to receive broadcasting, irrespective of the actual costs of delivering the service, was the foundation of the public service. The universal licence fee freed it both from the damaging limitations of commercial advertising revenue, and direct dependence on state revenue. It wasn’t a business and it wasn’t a department of state. It served citizens wherever they lived, was fundamentally redistributive and was not focused on well-off or convenient markets as a commercial service would have been. It was also a moral and political project that was in the national interest.^1^ The capacity to serve everyone was implicit in the technology. John Reith, the BBC’s first Director-General, was almost alone in grasping this.

In 1926 the British Broadcasting Corporation was given a mission to ‘inform, educate and entertain’ the public. Coinciding with the final extension of the franchise to young women, it was created out of an anxiety with the impact on democracy this newly enfranchised electorate might have. And it was created out of the distrust of politicians left by the First World War, and the distrust of big business that the economic failure after the war created. There was good evidence of Russian attempts to intervene (rather clumsily then) in UK affairs, and official concern over public order.

The creation of the BBC came at a moment in time when there was more institution-building going on: public organizations tasked with rolling out other public utilities—water, roads, education—in the national and individual interest were set up all around. This wave was in some ways a forerunner of the United Kingdom’s unique National Health Service, which was founded in the wake of the Second World War.

The forging of the BBC occurred while the first (and last) General Strike in 1926 took place, a deeply divisive period in British society. The notion of ‘balance’ in reporting had to be invented—there was no template to relate to both sides during a conflict within the nation. In an inevitably imperfect way, arguably too much on the government side, the BBC creatively found a national audience by giving both the views of the striking miners (whose pay was being cut) and at least some of their establishment supporters a voice, as well as the government: this in the face of fierce government opposition when society was painfully divided. The BBC learnt a crucial lesson that both sides listened to it because it gave them accurate information. Within three months the BBC—listened to in gatherings of people congregating around the new and as yet few ‘wireless sets’—had a national audience. The lesson was clear: in order for people to listen you had to give them something they needed.

Astonishingly radical at the time, its aim was not to push voters in any direction, but rather to help them make better choices and live richer lives. In the eyes of the BBC pioneers, broadcasting was an inherently democratizing and life-enhancing public service. The very best of music and theatre, public discussion and a ringside seat at great events were to be part of every listener’s life. This was not an obvious or necessary role for broadcasting. In Germany during the 1930s, the same technology was employed as a very effective agent of Nazi nation-remaking.^2^ In America, a strong public service voice has never emerged as the market was seen as supreme.

Above all, the BBC was built on a progressive vision of an educatable, decent, aspiring public whose understanding (and pleasure) in many things could be made deeper and enhanced. Fun, feeling and irreverence soon became part of the programming offer. At its best the BBC came to follow John Stuart Mill, the British philosopher, in believing that however self-evidently true an argument seemed ‘if it is not fully, frequently and fearlessly discussed, it will be held as dead dogma not living truth’ (Mill 2010 [1859]: 214). The BBC was built on but also evolved a set of principles that would guide its governance and its operations:

- it was not a state broadcaster but the state facilitated it;
- it had an independent source of finance (the licence fee) that governments might set but which the BBC had the right to spend and invest as it thought fit;
- it was impartial, balanced and was editorially independent (a relatively complex issue over time but fiercely fought for and protected)^3^;
- it served the public interest and what the public were interested in;
- it would not provide the lowest common denominator of content—it was not in this sense commercial. It would offer the public things they did not yet know they liked. It was experimental.
- It would be impartial, but would do more than just seek ‘balance’.
- It would provide ‘popular’ programs that everyone could delight in as it belonged to everyone, everyone paid for it and in this way, it was accountable to everyone. ‘Good’ and ‘popular’ were not opposites. It was there to provide ‘public service popular’.
- As long as a reporter or program-maker followed these principles the organization would defend and protect them.

These principles emerged from exploring what it meant for the BBC to serve the public and what the novel concepts of editorial independence and impartiality might mean in practice. For instance, as the BBC interrogated the state on behalf of the public, it offered politicians a fair opportunity to explain their policies; but this did not prevent the BBC from coming into conflict with governments as they came and went.

The BBC as a nascent public institution was profoundly shaped by the first Director-General, John Reith, who oversaw the ‘start-up’ phase. He was a driven, bullying, difficult, wily, very tall and overpowering man who had been wounded in World War One. He had spent time in America improving the quality of the mass production of small arms. So, he understood the new opportunities of mass production and mass consumption. A Presbyterian Scot, his furious, gloomy Protestantism provided the new organization with the evangelical belief in the capacity of words, speech, (secular sermons) to transform lives and to save people and society. But he combined this with a brisk modernism: progress, engineering, women working (as they did for the early BBC). As a trained engineer he was able to put the BBC in the forefront of technological accomplishment. His ambition was boundless and his energy prodigious. He was also very effective in accomplishing the goals he had set for himself and the fledgling organization.

## A Very British Institution

The BBC has been a pillar of the United Kingdom’s peculiarly robust unwritten constitution. This flexible mish-mash of laws, customs, conventions and courtesies relies on the idea that politicians know where the unwritten lines of the constitution lay and never stray over them. ‘The British Constitution is a state of mind’ said the historian Peter Hennessy. It depends on a sense of restraint, politeness and sense all round to make it work. It depends on people obeying the fairness in the spirit of non-written conventions applied to new eventualities. It also depends on what you might call ‘system honor’: that it is shameful as well as imprudent to overstep the lines in the sand.

The BBC has a universalist, democratic responsibility to everyone in the United Kingdom. Alongside measurable data about them, the relationship comes from the public’s moods and needs: from the fun that diverts them and the arguments they want to have. This flexible settlement is now being tested in a radically different political and media landscape from the one in which the Corporation originally emerged.

The Corporation’s history has made the institution. During the Second World War it became indispensable to the British public: their reliable companion in anxiety and fear (and a warm source of distraction). In what was a war of national survival, there were inevitable compromises in independence. But as the BBC told reliable truths both at home and abroad, government came, grudgingly, to trust it, and delegated communicating to the public as a professional skill to the BBC. The BBC turned towards understanding audiences with a new urgency. Consequently, the BBC was able to register public frustration with the war in, for example, comedy shows like ITMA (It’s That Man Again), which ridiculed the bureaucratic controls of the war, or talks like those of J. B. Priestley which challenged official views and championed the public’s experience of the war. Crucially, while being anti-Nazi, it was not anti-German.^4^ Producers had an essential freedom to make programs that interested a war-weary audience. The BBC was made great in studios and in arguments about how to broadcast best: not in the meetings of the Board of Governors.

In occupied Europe—where people risked their lives to listen to it—it gave audiences accurate news when they could trust no other source. It did not flinch (much) from telling them how badly things were going in the United Kingdom and in battles. ‘Its greatest victory’, according to George Orwell, was its accurate news. ‘Even in India where the population are so hostile they would not listen to British propaganda and will hardly listen to a British entertainment program, they listen to BBC news because they believe it approximates to the truth’ (Orwell 1944). Telling the truth in as far as it could be ascertained embodied a moral authority in the BBC.

During the Cold War, broadcasting into closed Eastern Europe was a lifeline for populations starved of reliable information. It was trusted because it did not preach and concentrated on what audiences in closed societies needed to know and wanted. In the twenty-first century, as more societies suffer from closed, inadequate, intimidated information systems and media this is still (surprisingly) a vital task.

It was program-making that give the Corporation an unrivalled relationship to other aspects of national and community life. It has different data about the public than any other service: it has to provide programs for when people get up and when they are tired, over the year. And it has a direct relationship with people over the course of their lives: from childhood onwards, winding programs into everyday experience. The public has to be wooed and enticed. The Corporation’s capacity to mobilize attention, delight, amuse and enthral, allied with the values of impartiality, objectivity and the right to interrogate power, wherever it lay, has been eyed warily but also avidly by commercial rivals and politicians alike. The BBC sees it as a duty to represent and explore the temper of the nation, to show the nation (for bad and good) to itself and include the nation’s voices in the national conversation.

## Structures, Crises, Adaptations

The BBC survived as an institution because the habits and structures that held it were reiterated and continuously adapted in policies and documents. It has a Royal Charter, which arrives in a very nice box with a remarkable amount of gold and red ribbons. This quaint custom has political meaning: it decisively puts the BBC in a special relationship to the constitution, above any mere government and to the Crown.

It has been subject to a regular cycle of reviews of the BBC Charter and licence fee (carefully distanced from the election cycle so that the BBC is somewhat protected from becoming a political football during an election). The most recent review of the Charter in 2016 asserted that ‘The BBC must be independent in all matters concerning the fulfilment of its Mission and the promotion of the Public Purposes, particularly as regards editorial and creative decisions, the times and manner in which its output and services are supplied, and in the management of its affairs’ (UK Government 2016). These reviews are an important part of refreshing the BBC. In 2016, during Brexit arguments, the BBC was told it had to represent the ‘diversity of the Nation’ to the Nation.

There have been many large overhauls, investigations and reports as well, usually as the product of some crisis. Most have developed along two fault lines: a clash with the government over a political matter when the government of the day felt that the BBC has not treated it fairly (Suez in 1956, the reporting of Elections in 1972, the Iraq War). The other fault line has been prompted by commercial rivals, asking why the BBC was subsidized to make popular programs that competed with them.

Sometimes the BBC made mistakes, was out of touch, failed to deliver. BBC crises burn fiercely—not least because the BBC has box office appeal, and its competitors, the Murdoch-owned media the other press and online competitors, have powerful platforms to attack the BBC. Another aspect of the relationship between the public and the BBC has been collective—it is an expression of Britishness and valuable in other lives, not just your own. This sense of ownership and collective will is another source of the Corporation’s political independence and resilience.

Holding the state to account is more difficult post-Brexit. In 2020, the newly elected Johnson government refused to put up any ministers onto the flagship morning radio program *Today*—a unique exercise of raw power. This dangerous innovation fell as the Covid 19 pandemic crisis sent politicians scampering back to the BBC to reach the public. Nevertheless for the BBC belonging to the nation is harder when the nation is at a more fractious juncture, and when the government of the day’s governing style reflects or exploits those divisions.

However, the BBC has always had—and still has—a distinct role that sets it apart from other organizations: its task is to hold other institutions to account on behalf of citizens. It stands at an angle to everything else. Some of the ‘ values’ that it calls on are also distinct: for example, the morality of news is solely driven by a duty to the story. This is a hard value to embrace. During thirty years of ‘The Troubles’ (the violent conflict in Northern Ireland, 1969–1998), the BBC had to be exempted from the Constitution of Northern Ireland that obliged government institutions to ‘work to promote peace’. This would have limited the BBC’s capacity to report fully and accurately on dissent and indeed violence.

This single-mindedness is tempered by responsibility and the never perfect, but vital processes that secure verity: a story has to be as accurate as the BBC can make it. But the *story* matters above all else, it has to be pursued without fear or favour: a fierce, professional and sometimes unnerving purpose. While much that is called ‘news’ is merely a business proposition, using the vehicle of sensationalism to attract audiences, BBC news values instead *ought* to reflect public interest values. News may indeed expose and indeed ‘harm’ some subjects—but not for profit and only in the public interest.

The BBC’s institutional legitimacy rests on the way in which it exercises its duty of vigilance and service to the whole nation—not the government of the day, not just the bits of the public that are rich, or the ones advertisers are interested in, not the fashionable nor the powerful who have influence, not the groups whose views it agrees with, or those it likes, or those who speak loudly, but the whole baggy, untidy nation. Freedom of speech in this perspective is not seen as a market or a battle in which the loudest, most popular voice is seen as a justified winner. Rather, the BBC’s aim has been to open the ring of potential speakers wider, to find and privilege minority voices, and to see argument not as a battle but as an organic and aesthetic process of development and reflection. The BBC has always tried to get people from the widest possible spectrum of British society (and beyond) to listen to each other. It steers close in this way to models of deliberative democracy.

Establishing where and who these communities are is an ongoing search. Puzzling out what they like and need is revealed experimentally, as it were, by making programs, gauging their uptake and people’s responses to them. Of course, the BBC can get out of touch, understand wrongly, and have its own in-built prejudices. But across the decades it has demonstrated its willingness to be curious and open-minded, and its ability to adapt and change its programming. The BBC has often been accused of bias on one side of some debate or another. Often the accusations are wrong (and come from a jealousy of the BBC’s authority). But they can also be correct. It then is the Corporation’s interest to adapt and reform.

## Craft

Most institutions have ‘craft’ at their hearts, which is found in the daily habits and skills of institutional members. BBC ‘craft’ resides in the program makers: those who make drama, documentary, comedy and entertainment; those who create new things in pursuit of the institution’s purpose. Some of their ideas may fail—public service can test originality and creativity and take risks in attempting to find new public tastes. *Fawlty Towers* was a failure on its first outing, and even on the repeat. Only later did it achieve iconic status. Then more recently it has been called into question. *Fleabag* became a cult hit because it riskily but deliciously identified in public (and very funnily) a shift in mores, yet its success was not predicted. The ‘craft’ is also found in the daily editorial decisions of news (enshrined in the BBC’s Editorial and Producers Guidelines [BBC 2019]—a magnificent contribution to the demanding task of making decisions in fast-shifting situations). It is this craft and protecting it that is the task of governance, management and the institution.

 BBC craft also involves understanding taste (a complex matter to bring off in the public interest). Take classical music. The BBC is the largest patron of music in the United Kingdom and the Proms is the world’s largest and most adventurous music festival. The constraints of public service have been liberating: it has a duty to keep and refresh the classical repertoire, commissioning new music, keeping up standards of playing and performance, exposing audiences to world music, the British musical tradition, to new ways of playing and new performing stars. It has some sense of advancing the understanding of music and reflecting and enhancing musical life. It has to grow audiences and foster tastes. The most important audience at the Proms—a strange summer-long love affair with music in central London and now elsewhere in the country—are the people who pay the least, who stand in the arena and who care the most.

The BBC’s ‘craft’ involves imaginatively creating new ways of talking with people in drama and children’s programming, in comedy and music. This entails the risk to deliver, for example, a drama series that creates new compassion as well as absorption; new ways of identifying social and political movement (often done better through culture, really, than politics), and new ways of representing diverse groups to themselves and others. The key to it all is the friable stuff of creativity. The BBC has been re-made in the last five years. It was noticable that during the volatile ‘Black Lives Matter’ protests of 2020 in the middle of the pandemic it was able to address a range of issues from health to the legitimacy of statues in public spaces with a responsive schedule. It was agile and in sympathy with public concerns and needs.

While the BBC cannot preach to the public, its programs can enlighten them. For example, David Attenborough’s series *The Blue Planet* (2018) put plastic waste onto the world’s agenda. Pictures of birds attempting to feed their young bits of glittering plastic appalled and mobilized young people and governments. Pictures of the mysterious intelligence and deviousness of octopuses changed attitudes towards an alien but great intelligence. Attenborough has worked for the BBC since 1954 and though decades of path-breaking nature documentaries have helped make its reputation. Attenborough’s breakthrough series, *Planet Earth* (1979), a hymn to evolution in 12 episodes, was revolutionary: it was made with new specially developed technology, a new business model, a remarkable script, an utterly new way of organizing shooting and research, and was based on and indeed developed scientific knowledge. Such programs come from investment. They are very expensive. They also depend on profound knowledge both of science and of program-making and their impact comes from their authority—and the integrity and scientific and televisual eminence of Attenborough himself.

## Governance

The BBC has been sustained by a system of governance that has secured its independence *from* but maintained a strong relationship *to* the state. The issue is how the BBC manages that relationship, and how it responds when agents of the state seek to exercise influence on how it governs itself and how it operates. The shape of BBC governance has changed but there are continuities and these have so far largely been husbanded carefully.

Originally, the Board of Governors ‘were’ the BBC. They held the Corporation to account in the public interest. They appointed the Director-General who was the Editor in Chief. The principles on which Governors were appointed have shifted. Sometimes they were chosen for ability but also to ‘represent’, for example, women, working-class interests or regional views. At times, political parties and governments have believed (sometimes rightly, sometimes alarmingly) that they need a ‘better’ representation. Occasionally people have been appointed who appeared hostile, but then turned into adept defenders of the BBC.^5^

Governments have, so far, largely respected the BBC and have been cautious about attacking let alone wrecking it. However, quite small changes in the system of governance can have large, long-term and unpredicted effects. The quality of ministerial responsibility is a real concern. The BBC used to be in large departments like the Home Office—and so overseen by senior ministers of standing. At the time of writing it is overseen by a minor ministry whose recent ministers have tended to be more junior, of variable quality and have changed all too frequently. Thus, in recent times the BBC has not had the level of ministerial protection it used to have.

Then there is the vocal power of opposition to the BBC, which has grown. Some of this is because in times of social polarization the BBC has a harder task in balancing a wider and more disparate set of political views. And at times when the opposition is weak and politics is fractious and angry, the BBC is always in danger of appearing to *be* the opposition, rather than reporting on it. Some of the criticism against it stems from commercial more than political motives, however. The UK tabloid press, for example, sees the BBC as competition online. Very large Media organizations like News International also see the BBC as a threat and campaign and lobby against it. Also, in the changing technological landscape the competitive position of the BBC has altered beyond recognition. Netflix, Facebook and other online rivals are far larger in scope: the BBC now competes with the biggest businesses that the world has ever known.

In 2007 (in response to a crisis), the Governors were abolished and an external, more independent body, the BBC Trust, was appointed in their place. The idea was that it could review evidence and set targets for the BBC at arm’s length. After a deep institutional crisis over Jimmy Savile—a paedophile who had abused his position as a prominent and long-serving BBC presenter to engage in egregious acts of behind the scenes sexual abuse—the Trust was replaced again in 2017 by a new supervisory board located within the BBC. At the same time, more of the oversight of the BBC has gradually been ceded to external bodies: the National Audit Committee can look into BBC finances and some of the regulation has been sent to the media regulator OFCOM. The task of making BBC appointments said one recently retired senior civil servant is ‘surrounded by processes that are designed to protect and distance such positions from political interference', but ‘depend in the last instance on political propriety’.

Overall, the more recent response pattern to challenging issues and critical incidents has been repeatedly to overhaul the BBC’s governance arrangements. Such continual tinkering with governance structures is destabilizing. One ex-official, who had spent a decade setting up the BBC Trust, observed:

Although the BBC as an institution had had a central role in sustaining civil society. and while the intention is still explicitly reflected in the “public purposes”, in practice its importance in this respect is diminished. This is partly to do with the context in which it now operates, with a multiplicity of providers undercutting its central role – as reflected in lower reach and share of audiences. It is partly to do with sustained attacks, both on “mainstream media” in general and the BBC in particular, partly with sustained attacks from commercial rivals which have caught the ear of government, but also it is to do with all sorts of monkeying around on funding, regulation and governance. As a result, the BBC’s position feels less stable and its role less central. (Nicholas Kroll, 2019, personal communication)

## Leaders and Members

Institutions are not made by governance structures alone. People make institutions and they can sink them. Institutional leaders, in particular, have to interpret the work of institutions in the context of the shifting challenges of the times. In times of stress, institutions depend on the sense of rectitude, the vision, the agility and cunning or even the bravery of individual leaders.

BBC Director-Generals have been remarkable in a variety of ways. Hugh Carleton Greene (1960–1969) was the last journalist to leave Berlin in 1939, ran propaganda into Germany during World War II, created the shape of German broadcasting after it, ran a successful hearts and minds campaign in Malaya and brought Berlin Cabaret-style satire to the BBC in the 1960s. He helped lead a shift in public taste and made the BBC central to a new generation. Greene was a remarkable defender of freedom of speech and because of his pre-war experience in Germany very sensitive to racial politics. Charles Curran (1969–1977), the first Roman Catholic DG had had a wide experience in the World Service and was an innovator, but as the BBC came under political pressure the BBC needed a more public face. John Birt (1992–2000) infuriated BBC staff, but called the future of technology better than any other broadcasting leader, not least because (like Reith) he was originally an engineer. Mark Thompson (2004–2012) was an outstanding news organizer. Tony Hall (2013–2019), a gifted, decent man, steadied the BBC after the Jimmy Savile crisis. Hall nurtured a new program-making excellence within the BBC. Hall lead the BBC through the Covid 19 crisis with inspiring warmth: the Corporation responded by inventing new services (in education for home schooling, in health and local information) and supporting the decimated arts especially music and getting beside the UK public in innovative ways. Yet the job has become so political that director-generals rarely go at a time of their choosing.

Most people working in the BBC for most of its history have felt something like a dedication to its values (not all of the time, of course). The BBC also breeds gossip more than other organizations because it has a sceptical purpose. Dedicated staff members often moan about the imperfect machine in which they are expected to do their work. Indeed, hostility to the ‘managers’ is an important feature of the organization. A recurrent allegation holds that the institution is failing to live up to ideals that most BBC people take to heart. Such allegations can be corrosive if it goes too far—but they can also be very powerful inculcators of values.

People who have worked in the BBC recognize that it seems to have a life of its own. And they are proud of it. Indeed, in interviewing many past leaders and program makers of the BBC I was struck by the way in which they readied themselves to be held to account. ‘The BBC’ said one senior manager, in the middle of a BBC conflagration and watching a career being trashed by the media outside, ‘is like a yoghurt monster. It eats you up to survive’. That is to say an admirable, successful career within the BBC may not protect you during a crisis.

Indeed, one of the ways in which the BBC works is that individuals take responsibility for errors, which may be real, or may merely be perceived, but which have impact. Sometimes the mechanism is faulty—reporters who exposed an injustice that caused a problem ought not to pay for the row their reporting provoked.^6^ But most BBC crises happen in public—not least because the BBC newsroom (magnificently but appallingly for the people at the centre of a crisis) turns ruthlessly on the BBC itself. It is a point of honour that the BBC will be reported on as forensically as any other subject of the news. This is a unique feature of the Corporation: it is very difficult to think of any other institution which savages itself so comprehensively in public. It is one of the formal and informal ways the institution regulates itself.

Institutions develop a hive mind—a collective consciousness, analogous to the behaviour of social insects, in which a group of people become aware of their commonality and think and act as a community. This is a resource, which helps members of that community to understand shared purpose, values and limits. The BBC World Service has preserved editorial integrity because it is attached to and protected by what one of its directors called ‘the mothership of values’ in the BBC (Sir John Tusa 2018, personal communication; also see Tusa 2018). It informs, for example, the way a news film is cut (frame by frame); it helps to determine how far a comedy show, at a particular time of day, for a particular demographic of an audience, can go with jokes, or how to assess the significance of a political event, or whether or what a classical and national repertoire of music needs to contain (when the canon is under attack).

The BBC’s hive mind depends on autonomous, conscious and moral individuals making such choices. The BBC has inculcated its workforce with values by a rigorous selection process, by treating people reasonably well, and by the everyday discipline of practical decision-making in newsrooms and commissioning meetings, in cutting rooms and in drama commissioning and delivery. Working for the BBC has clearly been something like a vocation: many staff felt (and feel) they were working for a principle. One intriguing feature of this tremendously competitive and professional cadre is that even as individual careers are sometimes wrecked during political or cultural rows, they recognize the necessity of the institution to survive.

Like the UK Civil Service, the BBC has in recent decades moved towards a more managerial model. This has changed the nature of its leadership strata: more managers, not first and foremost producers in the traditional creative sense. If this is developed further, it would have two consequences. Firstly, the BBC would have more leaders who have not come through program-making decisions on their way to managerial roles. And secondly, the route to the top would include more external candidates. Both of these will affect the composition of the hive mind of shared values.

## Precedent as Compass

 History matters to institutions. What looks like a complete pattern of relationships and rights has in reality been assembled over time and in response to different and challenging circumstances. In the case of the BBC, apparently long-lasting principles have always been identified in specific circumstances. Do we put a man who has admitted bombing a British Minister on the screen so that viewers can make up their own minds about him as he is interrogated by the interviewer, or is this endorsing violence? Does the BBC use the word terrorist? Is it a meaningful word?

Within the BBC, its history is used as precedent. It turns to the past for a starting point in many formal negotiations but also during attacks and problems. The BBC also has the rich store of material to celebrate (the first broadcast, De Gaulle’s broadcasts during World War Two, the first broadcast in Europe from a Mosque in 1937) and reuse in programming. So, the formal processes of recording history have been carefully nurtured. But history is also part of a collective memory. Previous examples are used in training programs but, more importantly, can be used to chew over the past and learn where things went wrong. The history is weighty. It adds heft.^7^

An important part of the BBC’s history is the succession of men and women who have been in charge of it (see Seaton 2016). The BBC has a relatively visible Director-General and a prominent, eminent and well-connected Chair. This makes it unlike, for example, the National Health Service. The recurrent defenestration of DGs and on occasions both DGs and Chairs has been a mechanism fraught with potential political threat and very traumatic to live through. Extraordinarily painful, indeed traumatic, for the individuals at the heart of the events, so far it has been a swift way of producing change and visible responsibility within the BBC, perhaps an instance of the ‘ritualized scapegoat’ theory of executive succession (Bynander and ‘t Hart 2016). It is first and foremost an expression of the intensely political nature of running the BBC—a way of solving nasty problems and the refreshing and reviving of the organization. One that, of course, could be abused.

## Today’s Challenge: A Revolution in the Architecture of Understanding

The BBC is now the fourth largest news organization in the world. What does being such a big provider and finder of news mean? You may get ‘news’ from Facebook, online, twitter and it may *feel* free and abundant. People certainly can (and it is a democratizing opportunity) tell eyewitness stories from where they are, from what is happening around them and in real time using their phones.

Tested journalism, editorially holding power to account and reporting from other places (whether that is the town along the coast or on the other side of the world) is hard to do, expensive, important and threatened globally. It has been a challenge to all of the traditional news organizations—particularly those dependent on advertising revenue. News and news outlets first had their content stolen by the social media companies. Then they had (if they were dependent on advertising) their revenues stolen by them, as viral advertising drawn by their content, went to the platforms who stole the content. Finally, they had the attention of their audiences stolen, through digital harvesting of preferences. This has led to a new political settlement whose consequences we can begin to see but which we have few tools to combat. Political leaders and groups can now communicate directly with voters and consumers, bypassing the traditional media gatekeepers and their interrogations.

Journalists and reporting are threatened by regimes all over the world. Violence against reporters has increased. Oppressive regimes have been remarkably successful at closing down reporting.^8^ Even in large, apparently lively democracies like India the restrictions on reporting have been startling. Business concerns that have direct interests in government now own most of the media. The space for independent and critical voices has shrunk dramatically. The use of intimidation, online attack, direct physical violence, the use of regulation to strangle opposition voices and so on—is now common.^9^

The BBC, with its reporters, bureaus, experienced editorial judgement, values and sheer heft of journalism, is in this sense a global resource. But it faces extraordinary competition. The Chinese state has invested in news organizations across the world pursuing its own foreign and domestic policy agenda. American social media companies have unrivalled budgets and have persuaded policy makers that they are merely ‘publishers’ with no duty towards content. But without a way of identifying and pursuing stories we become more ignorant and more vulnerable to false views of the world.  The ‘heritage’ media are immensley influential in providing the material for online discussion—so we need more quality reporting.

Algorithms are programmed to give you more of what you want. They allow you to avoid alternative points of view and encourage silos of information and feeling. Viral advertising has turned out to be a dreadful model for running socially responsible discussion (Moore 2018; Moore and Tambini 2018).^10^ The architecture of the social media means that views and interests that you become interested in appear to come endorsed by your peers and social group. Misinformation spread unwittingly between social media users has the direct impact of personal contact. Distrust of other points of view can be reinforced by sometimes fabricated but shared personal contact.

Modernity has been characterized by the growing significance of externally legitimated and authoritative information. This is being overturned by the new communication engineering of the internet and social media. We are now living through a great, fast, potentially dangerous revolution in public understanding. Although it offers many advantages, it is rapidly undermining political and business models, as well as the authority of well-sourced and reliable information.

The accidental and sincere sharing of information that is wrong has blown away journalistic conventions. This misinformation is persuasive because it comes from personal contacts. Yet it may be wrong because it is not tested (indeed it has the status of information but is really the same as pre-modern rumour). It can be spread by malicious propagandists, or campaigns that are partisan, passionate, but ill-founded. It can also be prompted by deliberate attempts to interfere in systems of understanding—disinformation.^11^ Some of these can be foreign, whose disruptive tactics lead people to question the very systems that they depend on for life. We are in the middle of something like an information war. Few politicians seem to have the capacity to understand the re-engineering of understanding that is underway.

The BBC lies on the grand fault line of having the intention, resources, practices and independence to produce the imperfect yet strenuously achieved and vitally important information that is proper news. Much robust social science research over the last seventy years has shown that individuals are resistant to propaganda because their social position, class, work place, family, age and gender determine their views more effectively than the media. However, the new social media revolution has turned that research on its head.

The BBC has to work with the turbulence these new communication systems are producing. The whole project of a shared reality based on shared facts is threatened. The BBC has to adapt and change to these new realities and risks. The impact on *everything* by the shift to the social media and the driving of all attention and belief into narrow silos, fed, produced and groomed by the model of viral advertising (you only encounter more of what you like), is the biggest challenge to the BBC since its inception.

Yet, over the last thirty years the BBC has, to some extent been limited from innovating in this new world in the public interest. The BBC proposed a public service search engine, where the algorithms would drive you to wider evidence; it proposed something like Netflix, it wanted to make a public interest information space. But it was prevented from pursuing these innovations (although it did make the *iPlayer* and the market for it). UK national media policy, run by a good regulator, prescribed the preservation of commercial fairness (although with a narrow but important dedication to preserving public service broadcasting).^12^ This policy prevented the BBC from creating public service access to the internet.

The BBC, like many other democratic institutions, has to arrive at innovative solutions to the problems posed by communicating in the twenty-first century. It has to find its way creatively to where people are, even if its regulatory framework and institutional structure have not been robust enough for the new realities. Political impartiality supported by balance and rigour was a consequence of its mission. That this is an ongoing, imperfect process is part of the strength of the BBC.

But the BBC faces an unprecedented ‘stress test’ in the new realities of social media, and an atomized audience. In a new, more nationalist phase of policy-making which puts the national interest above commerce, there may be an opportunity to create a new remit for the BBC based on the same enduring values. Indeed, as we are clearly in something like an information war, the BBC may be a tool with the journalistic weight to make a difference and the international reach to help create new public information spaces. Policymakers will have to unleash this creative energy (Mair 2020).

## Conclusion: The Interconnectedness of Decency

The propriety of institutions is contingent. Any one institution, probably unconsciously as well as formally, depends on the practice and strength of many others. They know each other indirectly and abut directly to each other, yet see themselves as distinct: the law and the civil service, the Monarchy or presidential systems, medicine, the universities and museums, the army and urban planning, parliament and local government. They subtly refer to each other, shape and condition each other, depend on and support each other. Their capacity to deliver their purposes and to identify improper pressure (and resist it) accumulates over time. It is hard to know what to do for the best in a crisis. All institutions face failures—they may let the public down; they may face unfair attacks. Even harder to identify is the slow insidious undermining of rules and propriety, but vigilance over little things matters.

The BBC is one of the institutions of the open society that potentially can be used on the side of reason and reliability. It has always dealt with emotional matters: drama and comedy, news and scandal, but the BBC also easily relates to the temper of the times. It is therefore especially fit to deal with the new age of fury and self-righteousness. Very few institutions have as part of their remit a direct responsibility to think about and help shape feelings creatively, but that is core BBC work.

The Corporation is a very peculiar beast. It has strong, complex ties to political institutions. It is also a public service that provides a binding capacity within the nation, an image of the nation to itself and an image of the nation abroad. And it survives—not merely as it were economically—but socially, constitutionally, politically, by amusing people and holding their attention for things that matter. It is peculiarly fragile. BBC history is often cyclical, the same problems appear again and again: but it is cyclical with terminal possibilities.

The BBC will survive if it creates magic: if it soldiers itself as it has in the past into people’s lives through moments and memory. While it has unique and appropriate powers and potential to call out abuse of power, it cannot stand alone in this endeavour. We look to well-functioning institutions to behave better than individuals do. Few of us can claim to be good, moral, people. We know how adaptable, safety-seeking and self-serving we are and how fallible our judgement. Not being heroes, we need institutions to call out the best in us and do better than we can. Professionally run institution help those who work within them as they can rely on the values, processes and purposes of institutions both for guidance and as a protection. Yet people that work in institutions must not be too obedient to the institution. Compliance and deference can lead to corrupt practices: as they have power institutions can abuse it. In some ways the BBC, full of stroppy-minded, professional hard-headed journalists ought to have some protection from such abuses.

Institutions like the BBC must not hoard power for themselves or only for their purposes. Nevertheless, they are more than the sum of their purposes and rules. Civilization is very complicated to sustain, and they make civilized life possible. Institutions are hard to build and take care of and pay attention. Yet they and the values that they hold in trust for citizens can be destroyed by carelessness or vandalism. The BBC has many features and values that make it poised for a new and greater role in contemporary life. Yet the propriety and power of institutions depends on their interconnected decency. The seventeenth-century British poet John Donne (1959 [1624]) wrote:

*‘No man is an island entire of itself: everyman is a piece of the continent,part of the main……the death of any man diminishes me because I am involved in mankindAnd therefore, never send to know for whom the bell tolls; it tolls for thee.’*

Perhaps no institution is an island.

## Questions for Discussion

1. Is having a national news institution like the BBC a good thing for a democratic society?
2. To what extent are all public institutions in the end dependent on political systems?
3. To what extent does leadership matter in explaining the institutional trajectory of the BBC?
4. In the present moment of technological, political and social turbulence, how can public institutions steer a responsible course? How can they help each other do the right thing?
5. ‘Perhaps no institution is an island’, so the chapter ends. What do you think is the author trying to tell the reader?

### Notes

1. There is a lively scholarly debate about which form was ‘best’: American historians prefer the market and European scholars prefer public service, see Hendy (2008), the work of Scannel (e.g. 1991), and the subtle work of Hilmes (1997). While both systems had advantages and innovated differently, and while ‘public service’ values reside in the American Press rather than broadcasting, America has failed to produce robust, sustainable, impartial public service broadcasting. A hundred years later the foundation of the BBC the commercialization of **all** communication clearly undermines democracy.
2. There is an extensive literature on the use of broadcasting by the Nazi state, most recently see Stargardt (2015) and Connelly et al. (2019).
3. The BBC remains the only organization, anywhere, of any kind that effectively sacked its own CEO on air (George Entwistle over the Savile affair 20).
4. Unlike the British public which did not like Germans. Yet such a distinction was essential for the post-war reconstruction of Germany. See Curran and Seaton (2018).
5. Chairs of the Governing body have often been appointed to ‘bring the BBC into line’. It looks and may be hostile. While Charles Hill (who had been Chair of the competitive Independent Television) was appointed Chair of the BBC by the Labour Government in this way, and was damaging, nevertheless, for example, Marmaduke Hussey and Christopher Bland were both initially controversial, seen as ‘politically’ appointed to tame the Corporation but in practice were excellent Chairs. Both oversaw a reorganization and growth of the BBC: they did not tame the BBC but helped creatively to change it’s direction. Too many recent Chairs have been business people—out of depth in public service.
6. Thus, Merion Jones who exposed Jimmy Savile’s abuse in a Panorama lost his job inexplicably. This caused legitimate resentment. It was also the case that 4 senior managers also lost their jobs. But they were responsible for the mishandling of Savile not indicting him.
7. The BBC first commissioned an official history when it was under attack during the Suez crisis. The history (written by Lord Briggs) was at first an outward facing defence. Now it is a recourse.
8. The author travels widely in South Asia talking to journalists and editors for a long-term UK project, and this is based on interviews with journalists from India, Pakistan, Bangladesh and Sri Lanka.
9. India slipped down the World Freedom of Press listings last year to an ignominious 140th place, down 2 from last year down 6 from 5 years ago.
10. Also see Moore’s excellent reports for The Centre for the Study of Media Communication and Power at https://www.kcl.ac.uk/policy-institute/cmcp.
11. See Watts (2018). But there have been excellent UK Government analysis and responses, see DCMS (2019).
12. The United Kingdom’s broadcasting regulator OFCOM has been excellent and has been a scrupulous protector of public service content more widely but having been tasked with preserving commercial fairness it has made decisions on that basis. The concern is always about ‘regulatory drift’ that the regulator is influenced by the industrial interests of the businesses it regulates.
